# An improved adaptive large neighborhood search algorithm for the flexible two-tier vehicle routing problem with drone stations

**DOI:** 10.3389/frai.2026.1759688

**Published:** 2026-03-13

**Authors:** Qi Li, Wenhao Yan, Tad Gonsalves

**Affiliations:** Department of Information and Communication Sciences, Faculty of Science and Technology, Sophia University, Tokyo, Japan

**Keywords:** two-tier vehicle routing problem, adaptive large neighborhood search, drone, last-mile delivery, mixed-integer linear programming

## Abstract

The two-tier vehicle routing problem (2T-VRP) represents a novel variant differing from the traditional VRPs. It can be applied to urban logistics operations system, offering significant potential for mitigating last-mile traffic congestion and reducing delivering costs. Distinct from traditional VRPs, this scenario contains a hierarchical two-tier structure, with trucks operating on the first tier and drones at stations on the second tier. In this study, we first extend the 2T-VRP framework by relaxing the hierarchical constraints, enabling trucks to transport goods not only to robot stations but also directly to customers. This new variant is referred to as the flexible two-tier vehicle routing problem with drone stations (F2T-VRP-DS). Then we formulate the problem as a mixed-integer linear programming (MILP) model. Finally, given the complexity of this problem, an improved adaptive large neighborhood search heuristic algorithm (IALNS) is proposed. The algorithm incorporates an adapted Clark and Wright saving algorithm as the initial solution, and a simulated annealing scheme is employed as the acceptance criterion. The experimental results show that our algorithm can provide high-quality solutions. In particular, compared with the MILP method, our algorithm demonstrates strong competitiveness on large-scale instances, offering smaller time consumption and better solution quality compared to commercial solver like Gurobi. In addition, based on the results of the sensitivity analysis, we further assessed the influence of several key parameters within the F2T-VRP-DS framework.

## 1 Introduction

The rapid growth of population and urbanization in recent years has created increasing challenges on last-mile logistics. In response, considerable research efforts have focused on improving delivery systems, with particular emphasis on multimodal delivery system as a key area of investigation. Multimodal delivery systems are developed to avoid the shortcomes associated with each transportation mode. In particular, truck operations are predominantly affected by factors such as customer accessibility, limited fleet size, traffic congestion, and infrastructural constraints, while other deliveries such as robots and drones are constrained by low travel speeds, limited storage capacities, and narrow temporal window (Campuzano et al., 2025).

However, the recent development of drone technology has provided new possibilities for last-mile delivery. Since 2010s, several well-known companies, such as Amazon, UPS, and FedEx, have been conducting experiments on drone delivery for the last mile (Thibbotuwawa et al., 2020). At the same time, governments worldwide have been actively engaged in formulating drone-related laws and regulations to standardize the management of drone operations. For instance, the European Union drone regulations, established by the European Union Aviation Safety Agency (EASA), came into force on January 1, 2021 (Alamouri et al., 2023). Similarly, the Chinese government enacted updated drone regulations, which became effective on January 1, 2024 (Gao et al., 2023). As previously noted, drones have advantages such as flexibility and efficiency, but their payload capacity and endurance are limited. In contrast, trucks have large payload capacity and expanded service coverage, but they are greatly affected by road conditions and terrain. The complementarity between trucks and drones makes their collaborative 2-tier delivery system a valuable research topic.

The structure of the classical two-tier delivery system is similar to a two-echelon distribution network. In this type of distribution network, trucks deliver goods from one or more depots to intermediate facilities, also called drone stations, which allow temporary storage of goods once they are unloaded from the trucks. Upon receipt of the goods, drone stations allocate the packages to drones, which then carry out deliveries to customers via multiple trips. When packages are delivered, customers can either receive the parcels in person or have them left at the doorstep unattended. To accommodate scenarios in which customers only accept in-person delivery, the delivery system needs to incorporate time window constraints, assuming that customers are at home only during specified periods.

The primary distinction between two-tier delivery systems and classical two-echelon delivery systems concerns the mode of second-tier delivery. In two-tier systems, drones are capable of making multiple trips between drone stations and customers, transporting a single package per trip. By contrast, in two-echelon delivery systems, drones can carry multiple packages simultaneously. Nevertheless, due to model complexity, second-tier vehicles in two-echelon systems are typically scheduled for a single deployment (Sluijk et al., 2023). Currently, the majority of existing research centers on the 2E-VRP. Nevertheless, urban delivery systems are characterized by high-volume, small-demand orders. According to a report by Meituan, over 60 million orders are fulfilled daily within their minute-level delivery network (Liang et al., 2024). Given the strict limitations on drones' battery capacity and payload, two-tier delivery systems may be a more suitable solution in urban environments.

In this paper, we introduce the flexible two-tier vehicle routing problem with drone stations (F2T-VRP-DS). Compared to the traditional two-tier or two-echelon vehicle routing problems, our model overcomes a out-of-date restriction of VRP formulations, where trucks are limited to delivering goods solely to stations and all customers must be served by second-tier vehicles. In our model, trucks can not only deliver goods to drone stations but also directly from depots to customers. This flexible delivery method is more reasonable and practical for real urban environments and helps reduce operational costs. To solve the F2T-VRP-DS problem, we first propose a mixed-integer linear programming (MILP) model. Moreover, we propose an improved adaptive large neighborhood search (IALNS) algorithm to solve larger-scale problems within a limited computational time. The main contributions of this paper are as follows:

(1) We propose a new variant based on the conventional 2T-VRP, which breaks the limitations of the traditional model by allowing trucks to deliver goods directly to customers while also transporting goods to second-tier stations. This flexible new model helps improve the convenience of the delivery system.

(2) We propose a mixed-integer linear programming (MILP) model to represent and solve the F2T-VRP-DS problem. In this paper, we use the latest commercial solver, Gurobi, to model and test this problem.

(3) We propose an improved adaptive large neighborhood search (IALNS) algorithm to solve the F2T-VRP-DS problem. Compared with the traditional ALNS algorithm, we improve the construction method of the initial solution and design several destruction and repair operators suitable for this problem. Moreover, a simulated annealing scheme and four local search strategies are employed to enhance the performance of the final solution.

(4) We perform sensitivity analyses on the proposed model and derive several insights concerning the enhancement of last-mile delivery efficiency.

This paper is organized as follows: Section 2 reviews the recent literature on the 2T-VRP. Section 3 provides a systematic description of the F2T-VRP-DS and formulates its MILP model. Section 4 presents the proposed IALNS algorithm in detail, while Section 5 analyzes the computational results and conducts sensitivity analyses. Finally, Section 6 concludes the study and outlines avenues for future research.

## 2 Literature review

This section presents a literature review of studies related to the F2T-VRP-DS. This study integrates the vehicle routing problem with drones (VRPD) and the two-tier vehicle routing problem. Therefore, we first review the VRPD, and then we discuss the current research on the two-tier/two-echelon vehicle routing problem.

### 2.1 Vehicle routing problem with drones

The Traveling Salesman Problem (TSP) and the Vehicle Routing Problem (VRP) are two classical problems in freight transportation and distribution. Over the past decades, they have been the subject of extensive academic research, leading to the development of numerous variants (Sluijk et al., 2023; Bogyrbayeva et al., 2024; Toaza and Esztergár-Kiss, 2023). With the development of drone technology and the increasing requirements for convenience and efficiency in urban delivery tasks, VRP and TSP models combined with drones have begun to attract attention (Rojas Viloria et al., 2021). The first drone-related freight transportation model was proposed by Murray and Chu (2015), known as the FSTSP model. It introduced an optimization problem in which a single truck and a single drone perform deliveries synchronously, with the objective of minimizing delivery time while satisfying all customer demands. The authors proposed a savings heuristic for this model, and the results showed that compared with the case of using only trucks, the new model can significantly reduce delivery time. The model soon gained attention. For example, De Freitas and Penna (2020) proposed a hybrid heuristic that first obtains an initial solution via a mixed-integer programming model and subsequently refines it into an FSTSP solution using an improved heuristic procedure.

In addition, researchers have further studied the application scenarios and constraints of the TSP-D. For example, El-Adle et al. (2023) proposed a new model that allows drones to be launched and retrieved at different locations. Tamke and Buscher (2023) studied the impact of drone speed on the delivery range. Jeong et al. (2019) and Cho et al. (2022) investigated the energy consumption of trucks and drones in the TSP-D model. The above studies mainly remain in the case of one truck equipped with one drone. In recent years, scholars have also begun to consider the TSP-mD model with one truck equipped with multiple drones. For example, Tu et al. (2018) designed an Adaptive Large Neighborhood Search (ALNS) and a Greedy Randomized Adaptive Search Procedure (GRASP) to solve the TSP-mD, and evaluated their performance on instances of different sizes. Their experimental results showed that ALNS can provide solutions with lower cost. Murray and Raj (2020) proposed a last-mile delivery system based on the TSP-mD, focusing on variables caused by practical factors such as drone endurance, payload capacity, and speed. In addition, they considered drone speed as a decision variable in order to minimize the delivery time.

In addition to the TSP, researchers have also investigated the VRP-D. Unlike the TSP-based variants, the VRP-D involves multiple trucks operating in coordination with drones, thereby substantially increasing the problem's complexity. Sacramento et al. (2019) introduced a VRPD model where each truck is equipped with a single drone that can take off and land when the truck is stationed at a customer node. This formulation considerably simplifies the problem and has become a foundation for numerous subsequent studies. Kuo et al. (2022) extended the model derived from Sacramento by considering the effects of time window constraints and designed a metaheuristic algorithm to solve the proposed model. In the same year, Huang et al. (2022) proposed an Ant Colony Optimization (ACO) algorithm to solve the VRPD problem. They also investigated two different scenarios, in which each drone serves either a single customer or multiple customers.

Although the single-drone VRPD is already highly complex, researchers have also investigated its multi-drone extension, the VRP-mD. For instance, Wang et al. (2019) examined a VRP-mD formulation in which each drone is restricted to carrying a single package. Rave et al. (2023) investigated a VRP-mD model incorporating intermediate stations, where drones are restricted to taking off and landing at predetermined locations. This model is to some extent close to the two-tier VRP studied in this paper, and they also examined the cost associated with using drone stations. In addition, Wang and Sheu (2019) also developed a multi-drone VRP model with intermediate stations, but in their model, drones can also be launched from the depot.

Truck–drone cooperative delivery models have also attracted increasing attention in real-world applications (Zhou et al., 2025). For instance, Mahmoudi and Eshghi (2025) proposed a multi-visit split delivery vehicle routing problem with drones to support post-disaster relief operations. Li et al. (2025) introduced the Vehicle Routing Problem with Drones and Variable Service Time (VRPD-VST) and validated the model using real agricultural virus monitoring datasets. In addition, Lyu et al. (2025) investigated a new variant of a cooperative rider–drone problem aimed at improving food transportation in rural areas.

### 2.2 Two-tier and two-echelon vehicle routing problem

In addition to the cooperation between trucks and drones as multiple transportation modes, this study also focuses on two-tier delivery systems. Therefore, it is necessary to introduce the recent research on two-tier/two-echelon VRP problems. The two-echelon VRP (2E-VRP) emerged from the recognition that, in contrast to the classical VRP, structuring the distribution network into two hierarchical tiers can frequently provide operational advantages (Sluijk et al., 2023). At the beginning, vehicles of different tiers were referred to as urban vehicles and city freighters, which were respectively used for pickup and delivery operations at depots and customer points. The two tiers of vehicles were connected through intermediate facilities called satellites, where goods were transferred and handed over. The initial definition of the two-echelon vehicle routing problem (2E-VRP) was provided by Crainic et al. (2009), who explored multiple variants of two-echelon distribution networks. The terminology 2E-VRP was subsequently introduced by Perboli et al. (2011), who formulated a mathematical model for the single-depot setting. Within the 2E-VRP framework, first-tier vehicles transport goods from a central depot to satellites, whereas second-tier vehicles are responsible for last-mile distribution to customers.

At present, two-echelon networks are increasingly applied in urban logistics, as they effectively address the challenges posed by traffic conditions, safety considerations, and the growing demand for clean energy. Consequently, researchers have explored multiple variants of the 2E-VRP, incorporating additional constraints tailored to the specific requirements of urban distribution tasks. Yu et al. (2020) investigated the 2E-VRPR-TW, a two-echelon VRP with time window constraints, in which each customer is assigned a fixed time window to capture real-world scenarios such as customer availability. Their sensitivity analysis revealed, in particular, that increasing the speed of second-echelon vehicles yields only marginal improvements in delivery efficiency. Chen et al. (2021) proposed the vehicle routing problem with time windows and delivery robots (VRPTWDR). While this formulation is not strictly a two-echelon VRP, its operational paradigm closely resembles that of the 2E-VRP. Their experimental results showed that the approach achieves robust performance in large-scale instances of up to 200 customers and proves highly competitive in densely populated urban areas with limited parking availability. Heimfarth et al. (2022) introduced the Mixed Truck and Robot Routing Problem (MTR-RP), where robot storage facilities and delivery sites are distinct. In this setting, trucks collect robots from specific depots and deploy them at separate locations to serve customers. Their analysis indicated that the MTR-RP achieves up to 40% cost savings compared with truck-only delivery models.

Unlike the 2E-VRP, the two-tier VRP (2T-VRP) operates by having trucks transport goods to designated robot or drone stations, from which the robots then perform the last-mile deliveries to customers. Poeting et al. (2019) analyzed a case study based on Cologne, Germany, and demonstrated that the placement of robot stations plays a critical role in distribution efficiency. Their results revealed that situating delivery stations in the city center can reduce the required number of robots by 25%. Alfandari et al. (2022) proposed the Uncapacitated Routing and Scheduling Problem (URSP), in which a single truck serves multiple hubs, and these hubs schedule robots to perform delivery tasks. They presented a MILP formulation and studied three variants of the URSP.

### 2.3 Research gap

In the previous review, we identified a research direction, namely the two-tier vehicle routing problem. This type of VRP focuses on mixed delivery with trucks and drones or robots, which can effectively meet the distribution needs of urban environments.Yet, most existing studies in this area conceptualize truck and drone operations as two separate systems, in which trucks merely transport goods to drone stations while drones handle all customer deliveries. Although such a formulation simplifies the problem, it fails to capture real-world complexity, particularly given the payload and range limitations of drones. In practice, urban logistics would benefit from allowing trucks to participate directly in delivery alongside drones. This more flexible mode of operation can better satisfy heterogeneous customer demands while reducing costs. To this end, we propose the F2T-VRP-DS model, wherein trucks perform both transportation and delivery functions in coordination with drones.

## 3 Problem description and formulation

### 3.1 Problem description

The F2T-VRP-DS characterizes a distribution system where trucks depart from a central depot, transport goods to drone stations, and simultaneously perform part of the customer deliveries themselves. Drones, in turn, are launched from the stations to execute round-trip deliveries to customers. As a complex variant of the VRP, the F2T-VRP-DS requires precise modeling. To this end, we introduce the following assumptions:

(1) All customers can be served either by trucks or by drones.
(2) A set of trucks is available for delivery tasks.
(3) Trucks can either deliver a single parcel to a customer or drop off multiple parcels at a drone station at once.
(4) Each drone can carry only one parcel at a time.
(5) Parcels delivered by drones must satisfy the payload constraint of the drone.
(6) The distribution area contains one depot, a set of drone stations, and a set of customers.
(7) Each drone station is equipped with one or more drones.
(8) Each truck has a capacity limit.
(9) Each truck departs from and returns to the depot.
(10) Each drone departs from and returns to the same drone station.
(11) Drones are subject to a maximum flight range constraint.

**Figure 1:**
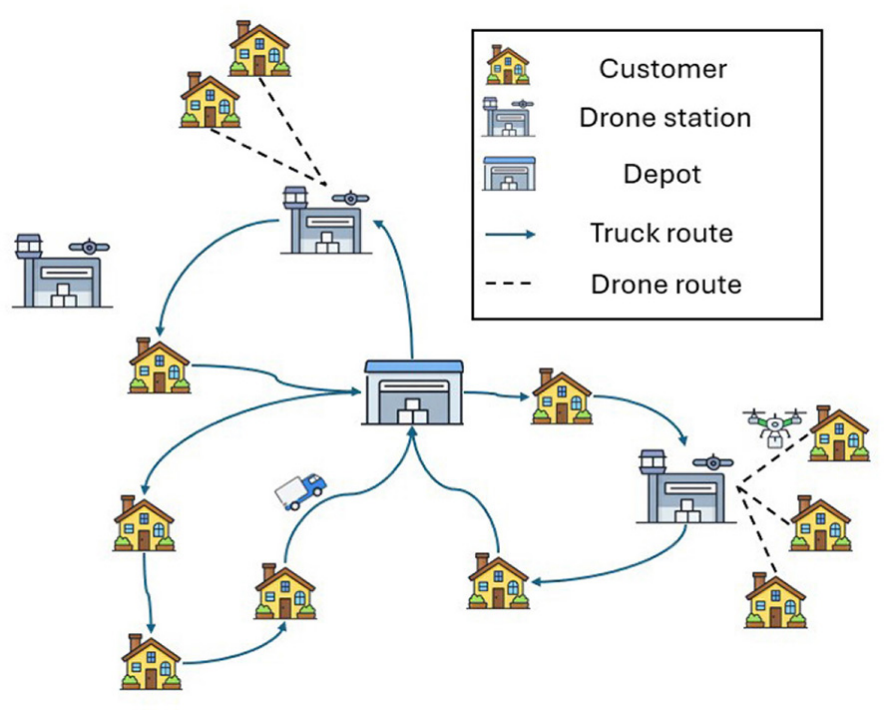
An example of F2T-VRP-DS distribution system.

Figure 1 illustrates an example of an F2T-VRP-DS distribution system. The network is composed of a single depot, three drone stations, and ten customer locations. In this instance, three trucks are dispatched from the depot to deliver goods to two drone stations and simultaneously serve six customers directly. The remaining four customers are assigned to drones, while one drone station is left unused.

### 3.2 Mathematical formulation for the F2T-VRP-DS

#### 3.2.1 Problem definition

The F2T-VRP-DS is defined over a directed graph *G* = (*V, A*), with node set *V* and an arc set *A*. The node set is defined as *V* = *D*^+^ ∪ *S* ∪ *C* ∪ *D*^−^, where *D*^+^ represents the starting depot, *S* is the set of drone stations, *C* is the set of customers, and *D*^−^ is the arriving depot. It should be noted that *D*^+^ and *D*^−^ are essentially the same location, and their positions coincide. Therefore, this setting satisfies the constraint that trucks must return to the depot after completing their routes. The arc set *A* = *A*_1_ ∪ *A*_2_ can be split into two sets of arcs for trucks and drones of the delivery system respectively. Hence, $A_{1}=\left\{\left(i,j\right)|i\in D^{+}\cup S\cup C,j\in S\cup C:i\neq j\right\}\cup \left\{\left(i,j\right)|i\in S\cup C,j\in S\cup C\cup D^{-}:i\neq j\right\}$, represents the arc set of the trucks route, *A*_2_ = {(*i, j*)|*i* ∈ *S, j* ∈ *C*} ∪ {(*i, j*)|*i* ∈ *C, j* ∈ *S*}, represents the arc set of the drones route. Table 1 illustrates the variables used in this mathematical model.

**Table 1:** Variables used in mathematical model of F2T-VRP-DS.

**Parameters**	**Description**
*x*(*i, j*)^*T*^	Binary variable, 1 if trucks travel from node *i* to *j*
*x*(*i, j*)^*D*^	Binary variable, 1 if drones travel from node *i* to *j*
*a*(*i, j*)	Distance from node *i* to *j*
*s* ^*T*^	Reciprocal of truck speed
*s* ^*D*^	Reciprocal of drone speed
$v_{s}^{T}$	Number of drone visiting times of each station *s*
$u_{i}^{t}$	Load of the truck at node *i*
$u_{s}^{d}$	Load of the drone at station *s*
*N*_*i*_	Demand of customer node *i*
*T*	Set of trucks
*D*	Set of drones

#### 3.2.2 Objective function

In this model, the objective is to minimize the total duration of all routes, including the truck routes and the drone routes (Equation 1). In contrast to the conventional objective of minimizing total traveled distance, our model explicitly incorporates the heterogeneous speeds of trucks and drones. The constraints of the model are defined in Equations 2–15.


$$\begin{array}{l} minF=\sum_{(i,j)\in A_{1}}x{\left(i,j\right)}^{T}a(i,j)s^{T}+\sum_{(i,j)\in A_{2}}x{\left(i,j\right)}^{D}a(i,j)s^{D} \end{array}$$ (1)

#### 3.2.3 Customer and station constraint

Constraint (2) ensures that each customer can be served by a truck or a drone. Constraint (3) specifies that for each customer, either one truck departs or one drone arrives, ensuring that every customer is served exactly once.


$$\begin{array}{l} \underset{j\in C}{\sum }x{\left(i,j\right)}^{T}+\underset{j\in C}{\sum }x{\left(i,j\right)}^{D}=|C| \end{array}$$ (2)

$$\begin{array}{l} \underset{j\in S\cup C\cup D^{-}}{\sum }x{\left(i,j\right)}^{T}+\underset{s\in S}{\sum }x{\left(s,i\right)}^{D}=1,\forall i\in C \end{array}$$ (3)

Constraints (4) and (5) enforce that each drone station is visited by at most one truck and that the number of drones departing does not exceed the station's maximum capacity.


$$\begin{array}{l} \underset{j\in S\cup C\cup D^{-}}{\sum }x{\left(i,j\right)}^{T}\leq 1 \end{array}$$ (4)

$$\begin{array}{l} \underset{j\in C}{\sum }x{\left(i,j\right)}^{D}+\underset{s\in S}{\sum }x{\left(s,i\right)}^{D}\leq |D| \end{array}$$ (5)

Constraints (6) and (7) establish a link between the number of drones departing a station and the number of trucks accessing it, thereby ensuring that drones cannot be deployed from stations that have not been visited by trucks.


$$\begin{array}{l} v_{s}^{T}=\sum x{\left(i,s\right)}^{T},\forall s\in S \end{array}$$ (6)

$$\begin{array}{l} \underset{i\in C}{\sum }x{\left(s,i\right)}^{D}\leq |D|v_{s}^{T} \end{array}$$ (7)

#### 3.2.4 Routing constraints

In this section, we define the truck and drone routing constraints respectively. Constraints (8) and (9) impose location restrictions on the routes of trucks and drones. Specifically, each truck is required to start from the depot and return to it upon completion of its route, whereas each drone must depart from a station and return to the same station.


$$\begin{array}{l} \underset{i\in D^{+}}{\sum }x{\left(i,j\right)}^{T}=\underset{i\in D^{-}}{\sum }x{\left(j,i\right)}^{T}=|T| \end{array}$$ (8)

$$\begin{array}{l} \sum x{\left(s,j\right)}^{D}=\sum x{\left(j,s\right)}^{D}\leq |D|,\forall s\in S \end{array}$$ (9)

Next are the flow conservation constraints for trucks and drones. Constraint (10) requires that the inflow and outflow of trucks at each node must be equal. Constraint (11) specifies that each drone must be launched from a station, serve exactly one customer, and subsequently return to the same station.


$$\begin{array}{l} \underset{\left(i,j\right)\in A_{1}}{\sum }x{\left(i,j\right)}^{T}=\underset{\left(j,i\right)\in A_{1}}{\sum }x{\left(j,i\right)}^{T} \end{array}$$ (10)

$$\begin{array}{l} \underset{i,j\in V}{\sum }x{\left(i,j\right)}^{D}=\underset{s\in Sj\in C}{\sum }x{\left(s,j\right)}^{D}+\underset{s\in Sj\in C}{\sum }x{\left(j,s\right)}^{D} \end{array}$$ (11)

#### 3.2.5 Capacity constraints

In our model, trucks are responsible for transporting goods to drone stations. Therefore, the model must not only satisfy the maximum capacity constraints of both trucks and drones (Equations 12, 13), but also ensure that the quantity of goods unloaded by a truck at each drone station equals the total quantity delivered by the drones departing from that station (Equations 14, 15).


$$\begin{array}{l} u_{i}^{t}\leq C^{T},\forall i\in D^{+},t\in T \end{array}$$ (12)

$$\begin{array}{l} u_{s}^{d}\leq C^{D},\forall s\in S,d\in D \end{array}$$ (13)

$$\begin{array}{l} \underset{t\in T}{\sum }u_{j}^{t}=\underset{t\in T}{\sum }u_{i}^{t}-x{\left(i,j\right)}^{T}N_{i},\forall i,j\in V,\forall j\notin S \end{array}$$ (14)

$$\begin{array}{l} \sum_{t\in T}u_{s}^{t}=\sum_{t\in T}u_{i}^{t}-\sum x{\left(s,j\right)}^{D}N_{j},\forall i,j\in V,\forall i\notin S \end{array}$$ (15)

## 4 Solution approach

In this section, we develop an improved Adaptive Large Neighborhood search algorithm (IALNS) to solve the proposed problem. Since the F2T-VRP-DS is an NP-hard problem, commercial solvers such as Gurobi are only capable of finding optimal solutions for small-sized instances. Hence, developing a metaheuristic algorithm is essential for efficiently solving large-scale problems.

The framework of ALNS was introduced by Ropke and Pisinger (2006) as a variant of LNS algorithm proposed by Shaw (1998). In recent years, it has been widely adapted in lots of new variants of VRP (Sacramento et al., 2019; Franceschetti et al., 2017; Chen et al., 2021). Similar to LNS, the ALNS algorithm follows the “destroy–repair” principle. It begins by generating an initial solution, then partially destroys it using a set of destroy operators, and subsequently reconstructs it with repair operators to obtain a new solution. The new solution is accepted if it improves upon the current one. In this study, we made several improvements to the traditional ALNS algorithm to enhance its performance. First, we developed a Clarke & Wright Savings algorithm to generate the initial solution. Second, we employed five destroy operators and three repair operators, each of which was modified to accommodate the requirements of the proposed problem setting. Third, we applied several local search methods to further refine the results. Finally, we adopted a simulated annealing mechanism as the acceptance criterion for new solutions. The pseudocode of IALNS is illustrated in Algorithm 1.

Algorithm 1 **The framework of IALNS.**

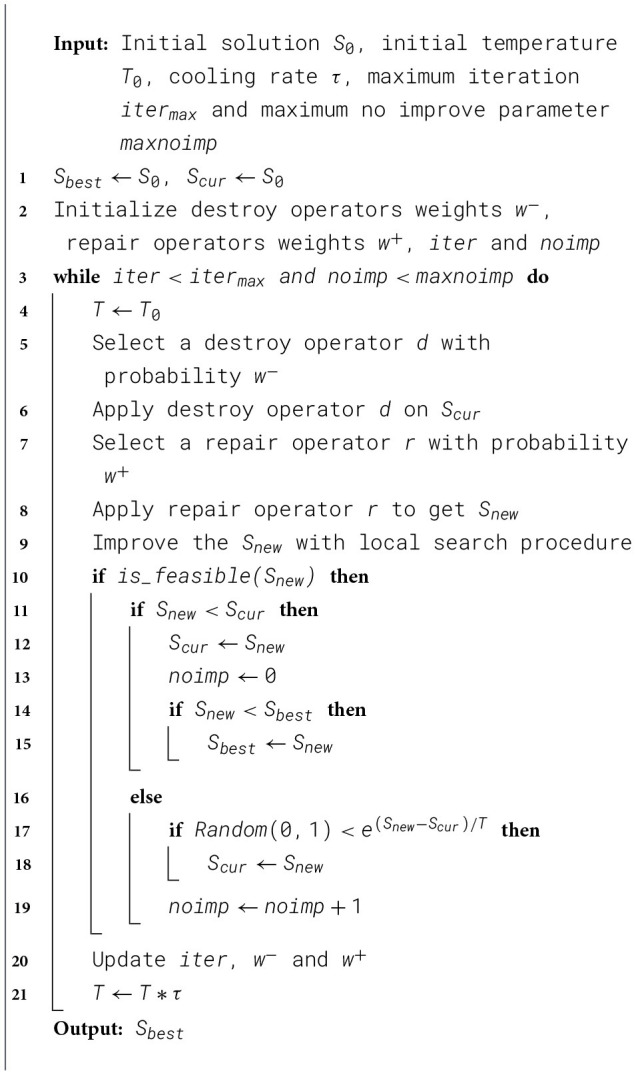

### 4.1 Initial solution

The first use of Clarke and Wright Saving algorithm in VRP is proposed by Altınel and Öncan (2005). Kong et al. (2025) firstly introduced this algorithm as part of the ALNS heuristic algorithm. In their research, a C&W Saving algorithm replaces the classic initial solution of ALNS. Inspired by their study, we developed an improved version of the Adaptive Clarke and Wright Saving algorithm (ACWS) as the initial solution for the F2T-VRP-DS problem. The specific workflow of the algorithm is shown in the figure below. We divide the workflow of the ACWS algorithm into the following steps. The framework of ACWS is illustrated in Algorithm 2.

Algorithm 2 **The framework of ACWS.**

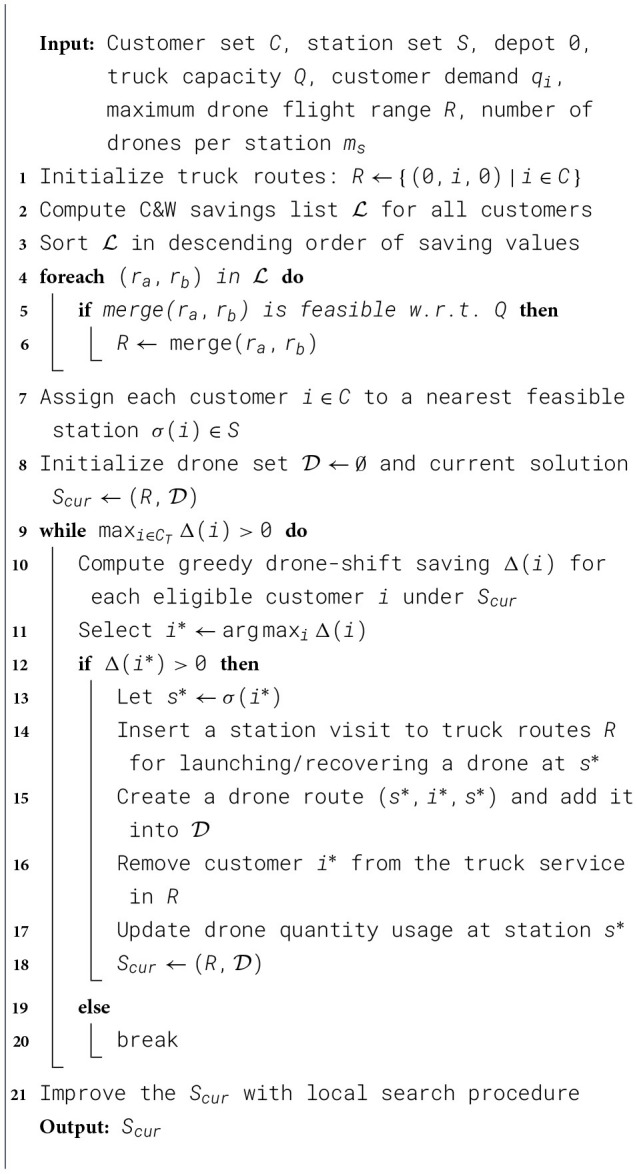

In the first step, we treat the problem as a classical Capacitated Vehicle Routing Problem (CVRP). Following the traditional Clarke and Wright savings algorithm, we first create one route for each customer, then calculate the savings obtained by merging each pair of routes based on the distance matrix, and finally merge the routes in descending order of their savings values. In this way, we obtain a CVRP solution.

In the second step, customers are assigned to the nearest drone stations according to the drones' maximum flight distance and quantity limitations. This step does not change the solution but provides a reference for inserting drone routes in subsequent stages.

In the third step, a greedy method is employed to evaluate the savings associated with assigning each customer to drone delivery. Customers are sorted in descending order of their savings values, and truck routes are progressively restructured—substituting direct truck deliveries with visits to drone stations from which drones undertake the service. Because each insertion of a drone route alters the truck network, the savings values must be recalculated each time a drone route is inserted.

In the fourth step, we apply classical neighborhood search methods such as swap, shift, and 2-opt to improve the overall route until no further optimization can be achieved.

### 4.2 Destroy operators

The next step of the IALNS algorithm is to use destroy operators to remove part of the existing routes. In the algorithm proposed in this paper, we employ multiple destroy operators, including both traditional ones and those specifically designed for drone stations.

(1) Random Removal (RR)
  The RR destroy operator operates by randomly removing *x* customer nodes from the current solution, with the value of *x* being positively correlated with the total number of customers.
(2) Worst Removal (WR)
  The WR operator identifies and removes the customer nodes that have the greatest impact on the objective function value.
(3) Shaw Removal (SR)
  The SR operator removes a group of related nodes. First, one customer node is selected randomly, and then several nodes that are most related to it are removed. The relatedness between nodes is calculated according to Equation 16:
$$\begin{array}{l} R\left(i,j\right)=\gamma d_{ij}+\theta |D_{i}-D_{j}|+\lambda g_{ij} \end{array}$$ (16)
  Since time windows are not considered in this study, the relatedness in the SR operator consists of three components: distance, demand, and route. γ, θ and λ are their weights, respectively. If customer nodes *i* and *j* belong to the same route, let *g*_*ij*_ = 1, otherwise, let *g*_*ij*_ = −1. The framework of SR is illustrated in Algorithm 3.
(4) Shark Removal (SKR)
  The Shark Removal (SKR) destroy operator works by removing a consecutive subset of customers from a single route. Compared to the previously introduced destroy operators, the SKR acts as a route-level operator, helping to disrupt the overall route structure and prevent the algorithm from getting trapped in local optima.
(5) Worst Drone Removal (WDR)
  As a two-tier VRP problem, we also design a dedicated operator called WDR to remove drone routes. This operator removes the worst-performing drone routes, including its originating drone station. It helps the algorithm reconstruct routes and avoid being trapped in local optima. The framework of SR is illustrated in Algorithm 4.

Algorithm 3 **Shaw Removal (SR) operator.**

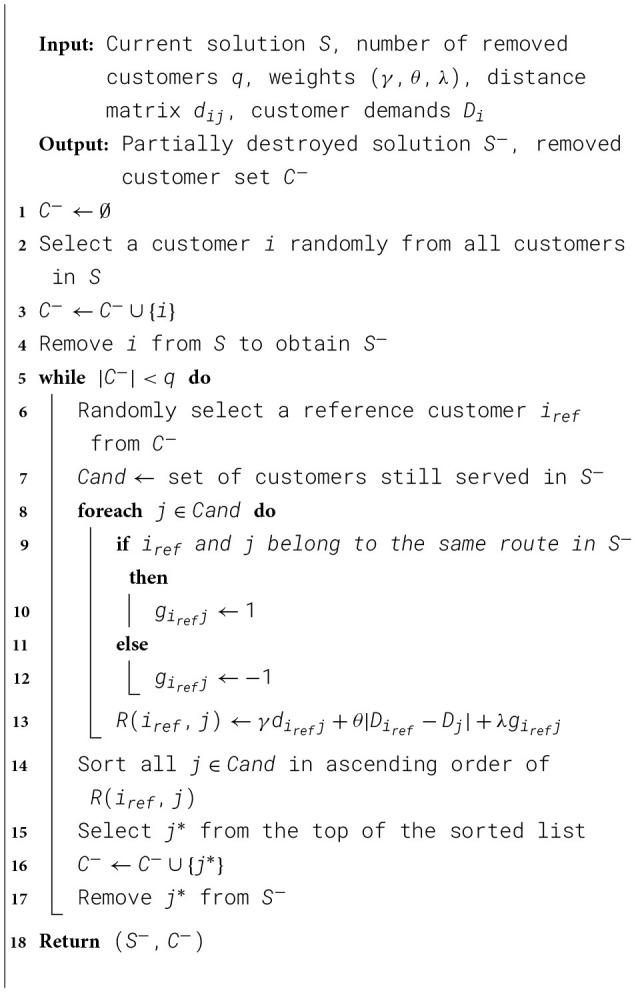

Algorithm 4 **Worst Drone Removal (WDR) operator.**

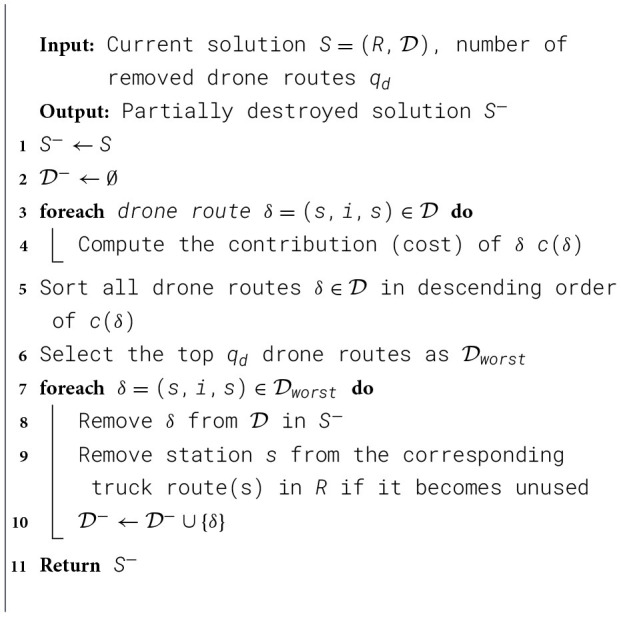

### 4.3 Repair operators

Our algorithm applies three repair operators. It should be noted that in our repair operators, drone route insertion is performed by first inserting the nearest drone station into the truck route and then generating the corresponding drone route. The operators must ensure that all customers removed by the destroy operators are reinserted into new routes.

(1) Greedy Insertion (GI)

The GI repair operator calculates the insertion cost of placing each removed customer into every feasible position. In this operator, the insertion costs for truck routes and drone routes are calculated separately. The selection of the drone's launch station is based on the nearest drone station assigned to each customer in the ACWS algorithm.

(2) Regret Insertion (RI)

The RI operator aims to select the next customer to be inserted based on forward-looking information. In this paper, we adopt the standard 2-regret method. That is, the regret value for each uninserted customer is calculated according to Equation 17:


$$\begin{array}{l} r_{2}\left(i\right)=c_{2}\left(i\right)-c_{1}\left(i\right) \end{array}$$ (17)

Here, *c*_1_(*i*) and *c*_2_(*i*) represent the increases in the objective value caused by inserting customer *i* into the best and second-best positions, respectively. In other words, the regret value indicates the potential loss if the customer is not inserted into its best position. Since the already inserted customers are no longer available, the regret values must be updated after each insertion.

(3) Best Drone Insertion (BDI)

This operator is inspired by the study of Chen et al. (2021). The removed customers are reinserted into the most suitable positions within the current routes, following a drone-priority principle. In other words, we first attempt to insert a drone station into the route and assign the customer to be served by that station, unless no feasible position is available. The framework of SR is illustrated in Algorithm 5.

Algorithm 5 **Best Drone Insertion (BDI) operator.**

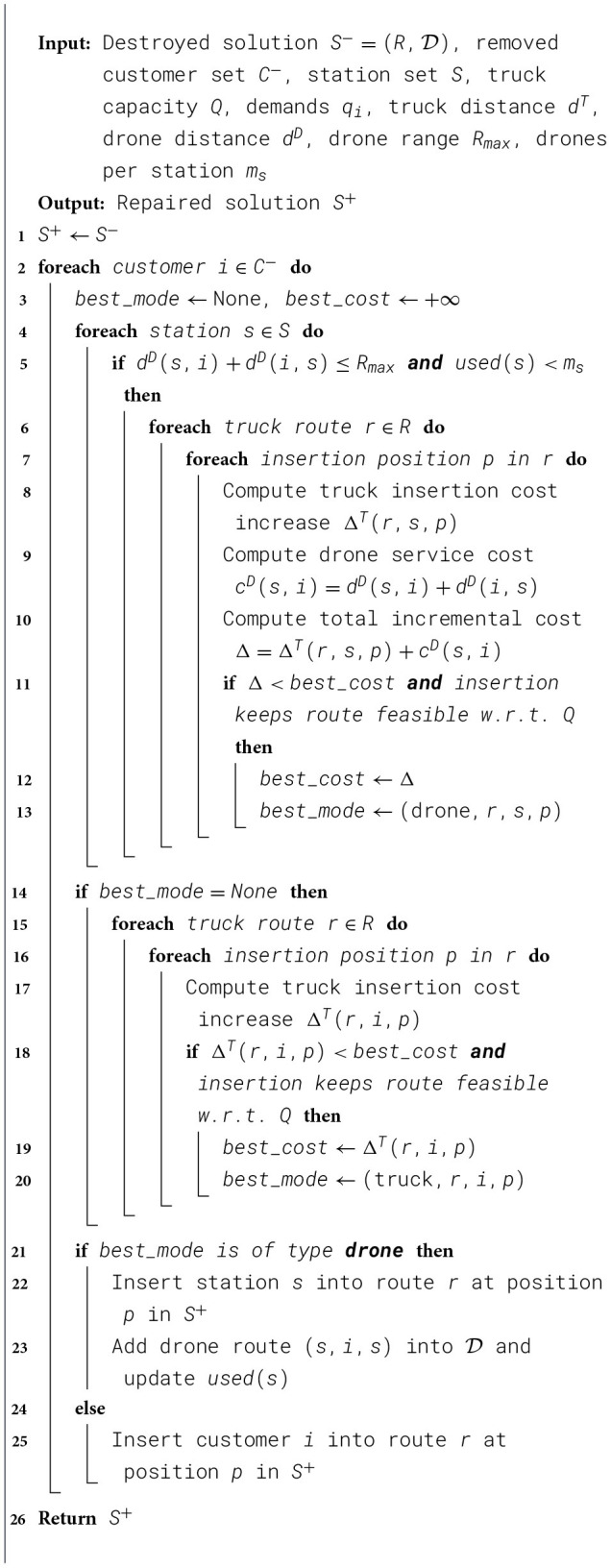

### 4.4 Local search procedure

To further optimize the results obtained from the IALNS algorithm, we introduced a local search procedure that includes four local search strategies.

(1) Removing duplicate stations: During the drone insertion process, it is possible for a truck to visit the same drone station multiple times. Since this study does not consider time window constraints, such repeated visits are unnecessary. Therefore, all repeated visits to the same station within a single route need to be merged into one.

(2) Intra-route 2-opt: For each truck route, a 2-opt local search is performed by removing two non-adjacent edges and reconnecting them in an alternative configuration. The process iterates until no additional improvement in the route length can be obtained.

(3) Inter-route swap: This strategy exchanges two nodes belonging to different routes. For customers served by drones, each customer is linked to a specific drone station. When a station is involved in the exchange, its corresponding drone route is simultaneously reassigned to the new truck route.

(4) Inter-route shift: This strategy relocates a customer from one route to another. Customers served by drones are excluded from this operation.

### 4.5 Acceptance criteria

We use a simulated annealing scheme as the acceptance criterion for new solutions. To avoid getting trapped in local optima, some new solutions that are not better than the current best solution can also be accepted. Specifically, when a new solution is worse than the current best one, it is accepted with a probability $e^{\left(S_{new}-S_{cur}\right)/T}$, where *T* denotes the temperature parameter. The temperature is initialized to *T*_0_ at the beginning of the search and subsequently reduced at each iteration using a cooling rate τ.

## 5 Computational experiments

In this section, we evaluate the performance of the proposed mathematical model and algorithm through a series of computational experiments. The test instances employed are adapted from the classical Solomon benchmark, which will be described in detail in Section 5.1. The algorithm was implemented in Python 3.10, while the MILP model was solved using the commercial optimization solver Gurobi v12.0.1. All the instances are solved on a computer with an AMD Ryzen Threadripper 3970x CPU.

### 5.1 Instance generation and parameter tuning

Since the F2T-VRP-DS is a new variant of the VRP, there are no available benchmark instances that match our problem setting. Consequently, we modified the classical Solomon benchmark instances to construct a suitable set of benchmark instances for our experiments.

Our instances follow the customer distribution patterns in the Solomon benchmarks: Random (R), Clustered (C), and Random–Clustered (RC). To accommodate the problem characteristics of the F2T-VRP-DS, a set of drone stations was additionally introduced into each instance. Specifically, based on the Solomon_100 dataset, we introduce additional drone stations at a ratio of 1:5 relative to the number of customers. The coordinates of the stations are generated randomly while attempting to ensure a uniform spatial distribution across the service area. For smaller instances, stations and customer nodes are selected proportionally at random, while all other settings remain unchanged. In addition, since this paper does not consider time window constraints, we ignored the start time, end time, and service time of customers in the Solomon instances.

The parameter settings used in our dataset are summarized in Table 2. The speeds of the trucks and drones are presented without specific units, since in the context of the two-tier VRP, the relative speed ratio rather than the absolute magnitudes determines the system dynamics. The effect of this ratio on overall performance will be systematically investigated in the subsequent sensitivity analysis.

**Table 2:** Parameter setting of F2T-VRP-DS.

**Parameters**	**Value**
Speed of truck	1
Speed of drone	2
Capacity of truck	200
Capacity of drone	50
Maximum range of drone	40

Table 3 presents the parameters of the IALNS algorithm. Different parameter settings are adopted for small-scale and large-scale instances. In this study, a pilot study is conducted to determine the parameter values. Specifically, several candidate parameter settings are first identified based on related literature (Che et al., 2022). These candidates are then evaluated through the pilot study, where both computational time and solution quality are jointly considered, and the best-performing parameter setting is selected. For small-scale instances, a minimum threshold is imposed to prevent the destroy operators from introducing insufficient perturbations to the routes.

**Table 3:** Parameter settings of IALNS.

**Parameter**	**Small-scale instances**	**Large-scale instances**
Initial temperature *T*_0_	1,000	1,000
Cooling rate τ	0.985	0.993
Remove rate *x*	0.2	0.2
Minimum number of removed customers	2	–
Minimum removal length of SKR	3	–
Maximum iteration	3,000	15,000
Maximum iteration with no improvement	200	500

### 5.2 Results of different scales instances

In this section, we evaluated the performance of IALNS algorithm. We compared the solution obtained by ACWS and IALNS with the MILP function obtained by Gurobi. Tables 4 and 5 summarize the computational results for small and large instances, respectively. In these tables, *obj* and Time denote the objective value and solving time. For the ACWS and IALNS methods, the *obj* values correspond to the average performance over 10 independent runs. *gap* indicates the difference between our IALNS algorithm and the MILP solution, while *imp* represents the improvement of IALNS over the ACWS algorithm. For the MILP results obtained by Gurobi, we set 3,600 seconds as the maximum computation time in order to obtain solutions within a reasonable time limit.

**Table 4:** Computational results of small-scale instances.

**Size**	**Type**	**MILP**		**ACWS**		**IALNS**				
		**Obj**	**Time(s)**	**Obj**	**Time(s)**	**Obj**	**Time(s)**	**Std**	**Gap (%)**	**Imp (%)**
5	C	166.14	0.07	229.17	0.0001	166.39	0.03	0.00	0.15	27.39
	R	143.94	0.08	196.74	0.0001	144.23	0.09	0.00	0.20	26.69
	RC	267.06	0.35	429.60	0.0001	279.95	0.19	4.80	4.83	34.83
10	C	290.76	0.42	350.72	0.0012	297.06	0.01	2.98	2.17	15.30
	R	219.53	0.20	295.34	0.0010	230.85	0.34	0.00	5.16	21.84
	RC	344.50	0.24	395.20	0.0001	358.42	0.21	31.91	4.04	9.31
20	C	460.97	45.33	644.43	0.0319	462.57	0.68	22.44	0.35	28.22
	R	266.00	3600	426.01	0.0020	286.97	0.03	3.91	7.88	32.64
	RC	436.78	36.79	625.55	0.0020	454.38	0.92	12.11	4.03	27.36
25	C	371.06	3600	632.23	0.0050	376.27	0.15	40.60	1.40	40.49
	R	291.17	3600	504.54	0.1700	260.73	0.73	11.15	-10.45	48.32
	RC	447.69	413.93	719.20	0.0020	443.07	0.96	13.37	-1.03	38.39
40	C	593.99	3600	805.15	0.0196	570.39	0.39	17.59	-3.97	29.16
	R	365.58	3600	497.95	0.0141	319.82	1.31	9.92	-12.52	35.77
	RC	746.45	3600	966.41	0.0441	674.35	1.43	28.37	-9.66	30.22
Average	–	360.77	1473.16	514.55	0.0196	355.03	0.50	13.28	-0.50	29.73

**Table 5:** Computational results of large-scale instances.

**Instance**	**MILP**		**ACWS**		**IALNS**				
	**Obj**	**Time(s)**	**Obj**	**Time(s)**	**Obj**	**Time(s)**	**Std**	**Gap (%)**	**Imp (%)**
c101_100	1243.84	3,600	1487.54	0.44	1100.95	17.59	51.48	–11.49	25.99
c102_100	1081.44	3,600	1362.53	0.25	1029.05	17.22	42.33	–4.84	24.48
c103_100	1315.82	3,600	1487.54	0.43	1066.18	19.37	39.67	–18.97	28.33
c104_100	1415.59	3,600	1494.61	0.28	1394.22	19.88	31.68	–1.51	6.72
r101_100	2200.67	3,600	2149.91	0.39	1601.04	50.73	107.48	–27.25	25.53
r102_100	2186.15	3,600	1646.61	0.25	1249.08	39.41	70.48	–42.86	24.14
r103_100	1865.95	3,600	1970.01	0.43	1610.42	40.52	92.22	–13.69	18.25
r104_100	1042.23	3,600	1085.73	0.04	796.80	81.17	53.80	–23.55	26.61
rc101_100	1021.66	3,600	1303.86	0.11	1001.04	25.87	68.52	–2.02	23.22
rc102_100	1093.41	3,600	1294.39	0.21	1057.50	49.97	34.65	–3.28	18.30
rc103_100	1247.79	3,600	1379.21	0.53	1145.37	51.65	69.41	–8.21	16.95
rc104_100	1337.10	3,600	1644.56	0.74	1161.94	70.96	22.59	–13.10	29.35
Average	1420.97	3,600	1525.54	0.34	1208.51	15.19	44.50	–14.23	22.32

Table 4 presents the comparative results for small-scale instances. It can be observed that, in small instances with only 5 or 10 customers, the MILP formulation can find the optimal solution within a very short time. However, as the instance size increases, the MILP model gradually fails to find the optimal solution within the time limit. Meanwhile, our IALNS algorithm performs slightly worse than the MILP in small instances but can also complete the computation within a short time. For relatively larger instances (e.g., those with 25 and 40 customers), the IALNS algorithm can obtain high-quality solutions superior to those of the MILP while maintaining high computational efficiency. In addition, compared with the initial ACWS solutions, IALNS achieves an average improvement of about 29.73%.

Based on the Solomon-100 dataset, we constructed an improved version containing 100 customers. The results are shown in Table 5. It can be observed that for all instances, the MILP formulation fails to find an optimal solution within the limited time. However, our IALNS algorithm can obtain solutions within 1 min. Moreover, the results of IALNS outperform those of the MILP by an average of 14.23%. In addition, compared with the initial ACWS solution, IALNS achieves an average improvement of 22.32%, demonstrating the effectiveness of the proposed algorithm.

### 5.3 Comparison the IALNS with GVNS algorithm

In 2024, Morim et al. (2024) developed a General Variable Neighborhood Search (GVNS) metaheuristic for a collaborative delivery model integrating trucks, drones, and robots. In this section, we conduct a comprehensive performance comparison between the proposed IALNS algorithm and the GVNS method. It should be noted that the problem setting addressed by the GVNS approach differs from that considered in this paper. Therefore, we introduce several modifications to adapt the algorithm to the F2T-VRP-DS setting.

First, the robot-based delivery policy in the original study is conceptually closer to the drone delivery policy considered in this paper. Accordingly, we remove the drone delivery component from the original VRPD-RS model and modify the robot delivery logic so that it is consistent with the drone delivery mechanism adopted in this study. Second, since the drone delivery component in the VRPD-RS model is removed, the synchronization constraints between drones and trucks, particularly those related to time window matching, are no longer required. To further adapt the algorithm to our problem setting, we also relax the restriction that each robot station can only be served by a single truck. Finally, our objective function is more closely aligned with the operational cost considered in the original study; therefore, we do not adopt makespan as the evaluation metric in our experiments. The remaining algorithmic design and parameter settings of GVNS are kept the same as in the original paper. We preserved the original neighborhood operator design of GVNS to the greatest extent possible. Although both two algorithms belong to the family of neighborhood-based heuristics, the neighborhood structures underlying ALNS and GVNS are fundamentally different; therefore, the operators designed for IALNS cannot be directly applied to GVNS.

Table 6 reports the comparative results of IALNS and GVNS on instances of different sizes. The parameter settings of ALNS are the same as those used for the small-scale instances in Section 5.2. The parameter settings of GVNS follow those in the original paper, where *k*_*max*_ = 4 and $n_{max}=n^{2}/k_{max}$, with *n* denoting the number of customers in each instance. Each result represents the average value obtained from 10 independent runs, and Std denotes the corresponding standard deviation. In addition to the objective value and computational time, we also report the number of drone deployments for both algorithms, denoted by *N*_drone_. As shown in the table, for the majority of instances, both algorithms produce satisfactory solutions within reasonable time limits, but the proposed IALNS algorithm outperforms GVNS in terms of solution quality. In addition, we further analyze the detailed characteristics of the solutions generated by the two algorithms. The results reveal that IALNS exhibits a higher level of drone utilization than GVNS, suggesting that GVNS relies primarily on truck-based deliveries. We believe that this more effective exploitation of drones contributes to the superior performance of IALNS compared to GVNS.

**Table 6:** Comparison between IALNS and GVNS.

**Size**	**Type**	**IALNS**				**GVNS**				**Gap (%)**
		**Obj**	**Time(s)**	**Std**	*N*_drone_	**Obj**	**Time(s)**	**Std**	*N*_drone_	
5	C	166.39	0.03	0.00	2.0	167.08	0.09	2.81	5.0	–0.41
	R	144.23	0.09	0.00	2.0	145.03	0.15	0.00	2.0	–0.55
	RC	279.95	0.19	4.80	2.2	288.11	0.08	0.00	5.0	–2.83
10	C	297.06	0.01	2.98	6.8	297.41	1.54	26.55	4.5	–0.12
	R	230.85	0.34	0.00	6.0	225.15	1.29	26.92	5.9	2.53
	RC	358.42	0.21	31.91	5.0	418.37	3.44	27.74	5.2	–14.33
20	C	462.57	0.68	22.44	12.4	461.47	10.59	34.29	6.5	0.24
	R	286.97	0.03	3.91	9.0	314.22	5.33	11.86	2.0	–8.67
	RC	454.38	0.92	12.11	10.2	450.13	5.71	18.42	5.2	0.94
25	C	376.27	0.15	40.60	12.4	507.83	14.01	40.02	1.9	–25.91
	R	260.73	0.73	11.15	9.0	379.13	9.56	8.13	2.6	–31.23
	RC	443.07	0.96	13.37	8.4	452.77	13.38	19.38	4.2	–2.14
40	C	570.39	0.39	17.59	22.0	813.93	95.81	36.64	6.9	–29.92
	R	319.82	1.31	9.92	13.0	585.44	41.47	32.27	1.6	–45.37
	RC	674.35	1.43	28.37	16.4	518.63	51.46	7.45	4.2	30.03
Average	–	355.03	0.50	13.28	9.12	396.79	103.16	25.53	3.04	–10.25

To further assess the statistical significance of the performance difference between IALNS and GVNS, a Wilcoxon signed-rank test was conducted on the paired objective values obtained from all tested instances. The null hypothesis assumes no significant difference between the two algorithms, while the alternative hypothesis states that IALNS outperforms GVNS in terms of solution quality. Let *d*_*i*_ = *x*_*i*_ − *y*_*i*_ denote the paired performance difference between IALNS and GVNS on instance *i*. After removing zero differences, the absolute differences |*d*_*i*_| are ranked in ascending order. The Wilcoxon signed-rank statistic is defined by Equation 18


$$\begin{array}{l} W=\overset{n}{\underset{i=1}{\sum }}R_{i}\cdot 𝕀\left(d_{i}>0\right) \end{array}$$ (18)

where *R*_*i*_ is the rank of |*d*_*i*_| and *I*(·) is an indicator function. Under the null hypothesis that the median of the paired differences is zero, the distribution of *W* is used to compute the corresponding *p*-value.

The statistical test results indicate that IALNS significantly outperforms GVNS at the 5% significance level in a one-sided Wilcoxon signed-rank test (*p* = 0.0365). The result indicates a clear and consistent performance advantage trend of IALNS across most instances. Considering the relatively small sample size and the heterogeneous characteristics of the benchmark instances, this marginal significance is reasonable and still provides meaningful statistical support for the effectiveness of the proposed IALNS framework.

### 5.4 Sensitivity analysis

This section presents a sensitivity analysis of several key parameters in the F2T-VRP-DS framework. All reported results represent the average values obtained from 10 independent runs.

#### 5.4.1 The number of drones per station

**Figure 2:**
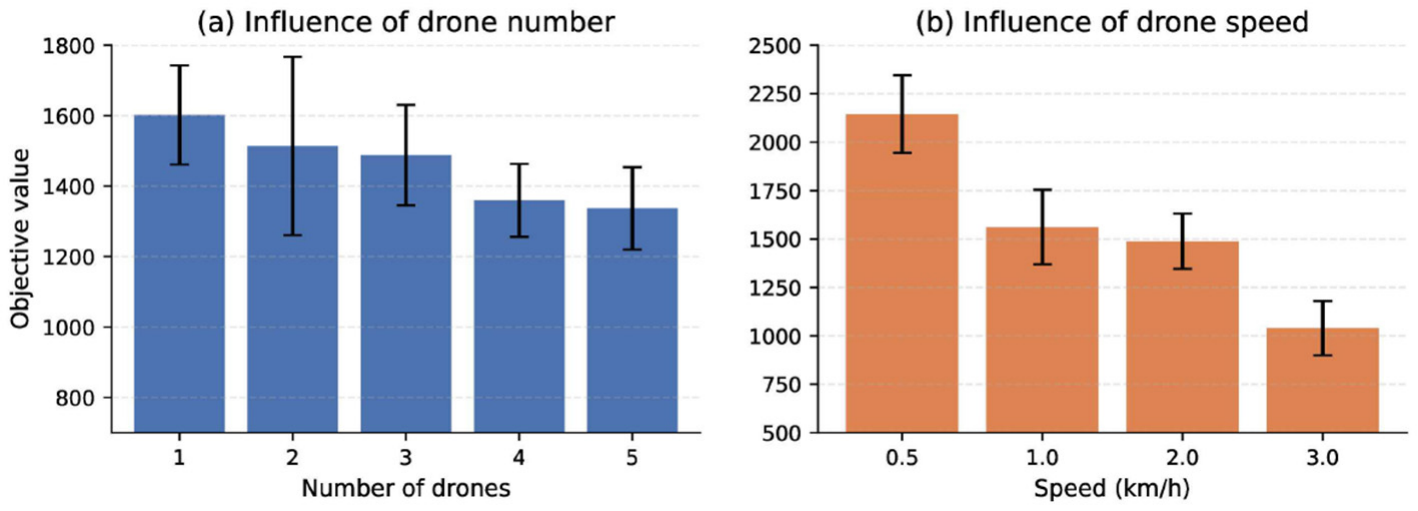
Comparison of the number of drones. **(a)** Influence of drone number. **(b)** Influence of drone speed.

**Figure 3:**
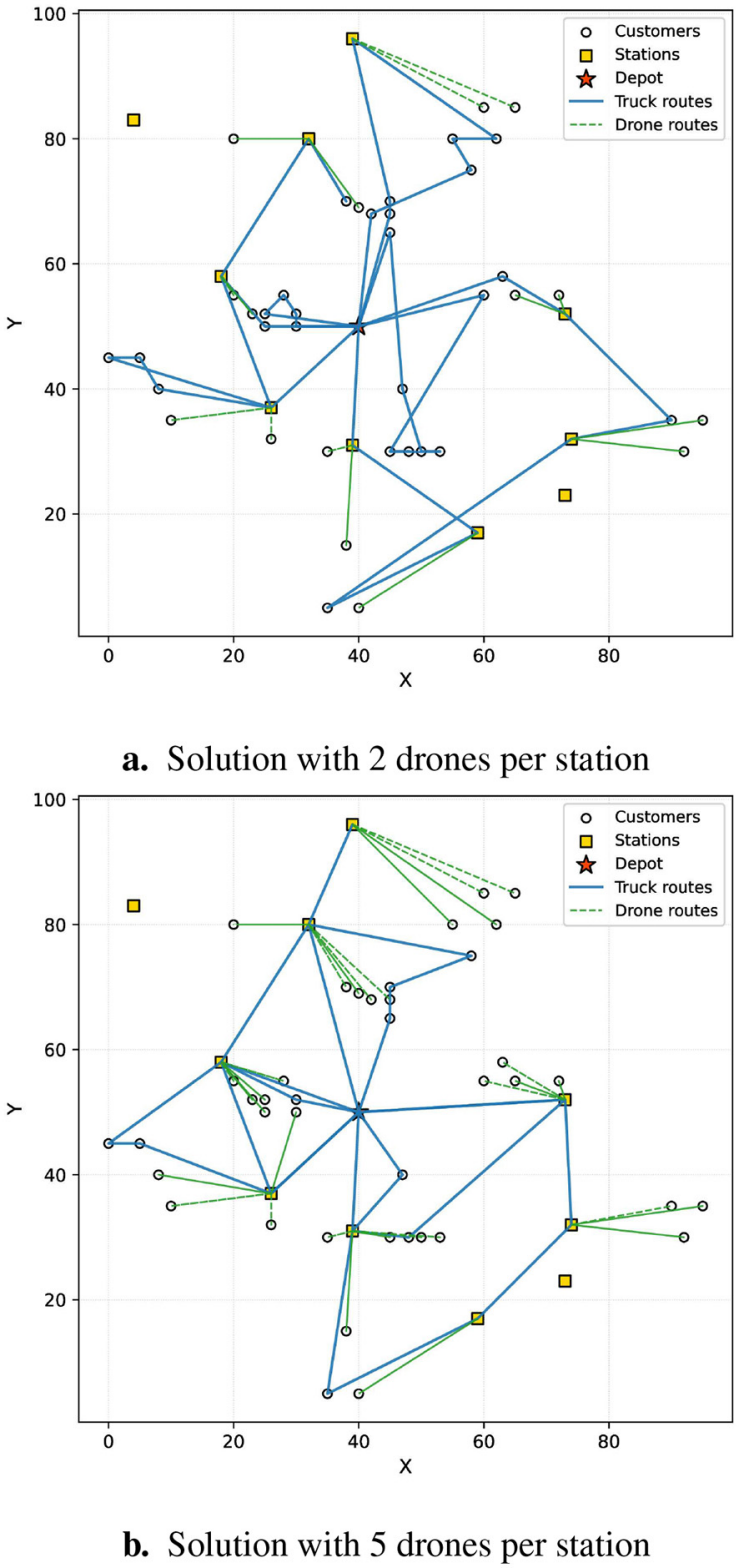
Example of different number of drones in 40 customers' instance. **(a)** Solution with 2 drones per station. **(b)** Solution with five drones per station.

First, we examined the influence of the number of drones assigned to each station on the overall performance. Figure 2a presents the average results for the 100-customer instance. The figure shows that the objective value gradually decreases as the number of drones per station increases. This trend arises because a larger drone fleet allows the truck to offload more delivery tasks to drones, thereby simplifying its route. Figure 3 provides a visual comparison of the routes obtained for the 40-customer instance under two configurations—each station equipped with two drones and five drones. The results clearly demonstrate that a larger number of drones leads to a markedly simpler truck route.

#### 5.4.2 The speed of drones and trucks

As we mentioned before, the relative speed ratio rather than the absolute magnitudes matters in F2T-VRP-DS. Therefore, in this section, we make a sensitivity analysis for the speed of trucks and drones. Typically, trucks travel at much higher speeds than drones. Nevertheless, in practical scenarios, truck movements are constrained by traffic conditions, while drones can travel in straight paths toward their destinations. Consequently, in our simulation environment, the drone speed is assumed to potentially exceed that of trucks.

From Figure 2b, we can see that the objective value decreases as drone speed increases. On the one hand, this is because the objective value represents the total time consumed by both drones and trucks, and higher drone speeds directly reduce this time. On the other hand, as drone usage becomes more cost-effective with increased speed, the algorithm tends to assign more deliveries to drones, which further reduces the objective value.

**Figure 4:**
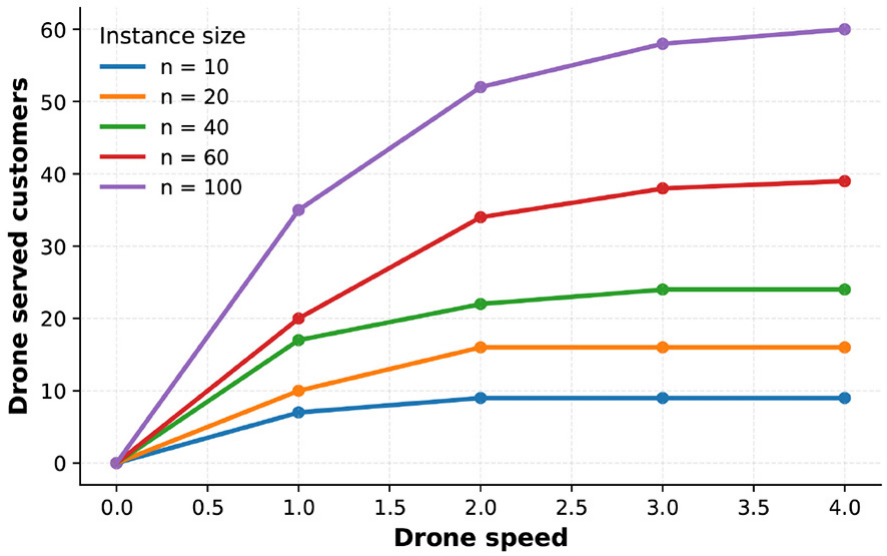
Comparison of the speed of drones.

We also evaluated the proportion of customers served by drones at different drone speeds. As shown in Figure 4, we calculated the number of drone-served customers for instances ranging from 10 to 100. When the drone speed is 0, no customers are served by drones, as the model degenerates into a classical CVRP. In addition, a pronounced diminishing marginal effect of drone speed is observed. For several of the tested instances, once the speed ratio between drones and trucks exceeds 2, the results stabilize, suggesting that continually increasing drone speed does not fundamentally change the solution structure. We believe that the underlying reason is as follows. When the drone speed is relatively low, the solution quality is primarily constrained by the drone travel time. Therefore, as the drone speed increases, the number of customers served by drones rises significantly. However, once the drone speed becomes sufficiently high, the proportion of drone-related cost in the total objective value becomes relatively small. In this case, further increasing drone utilization does not lead to a noticeable improvement in solution quality, and consequently, the curve of the drone service ratio tends to flatten.

#### 5.4.3 The capacity of drones

In previous studies, the payload capacity of drones was set to 50, which also corresponds to the maximum customer demand in the benchmark instances. However, in practical situations, drones may not be able to meet the delivery requirements of certain customers. Therefore, it is necessary to investigate the relationship between drone payload capacity and the proportion of customers served by drones.

**Figure 5:**
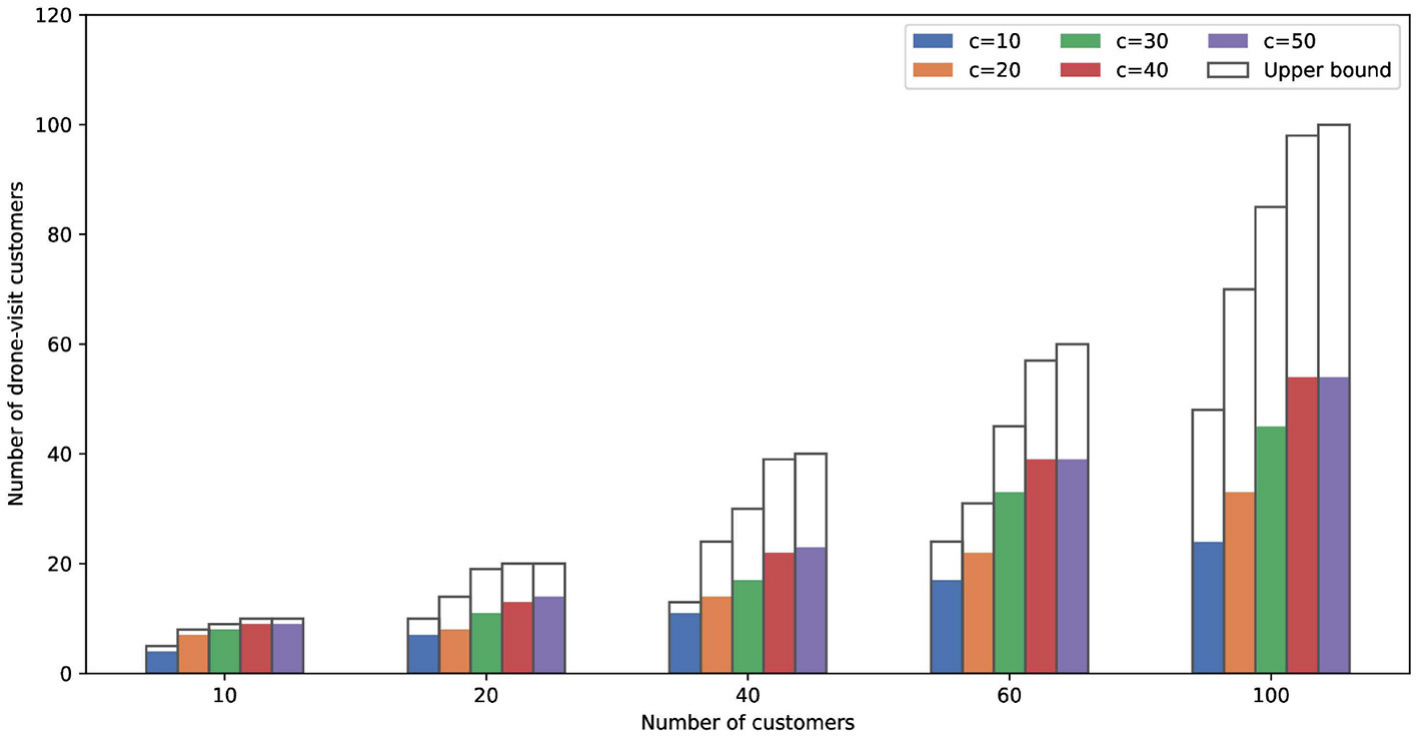
Comparison of the capacity of drones.

We examined the effect of varying drone payload capacities, ranging from 10 to 50 on the experimental results. Figure 5 presents the proportion of customers served by drones across instances of different sizes under various payload settings. The upper bound indicates the maximum number of customers that can potentially be served by drones. As illustrated, the number of customers serviced by drones increases as the payload capacity grows. Moreover, we observe that the proportion of customers served by drones decreases as the instance size grows. This is because the complexity of truck routes increases with the number of customers, making direct truck service a more favorable option than transporting parcels to drone stations. In addition, increasing the drone payload does not change this trend, indicating that drone payload capacity has only a limited impact on the overall routing plan.

### 5.5 Case study

**Figure 6:**
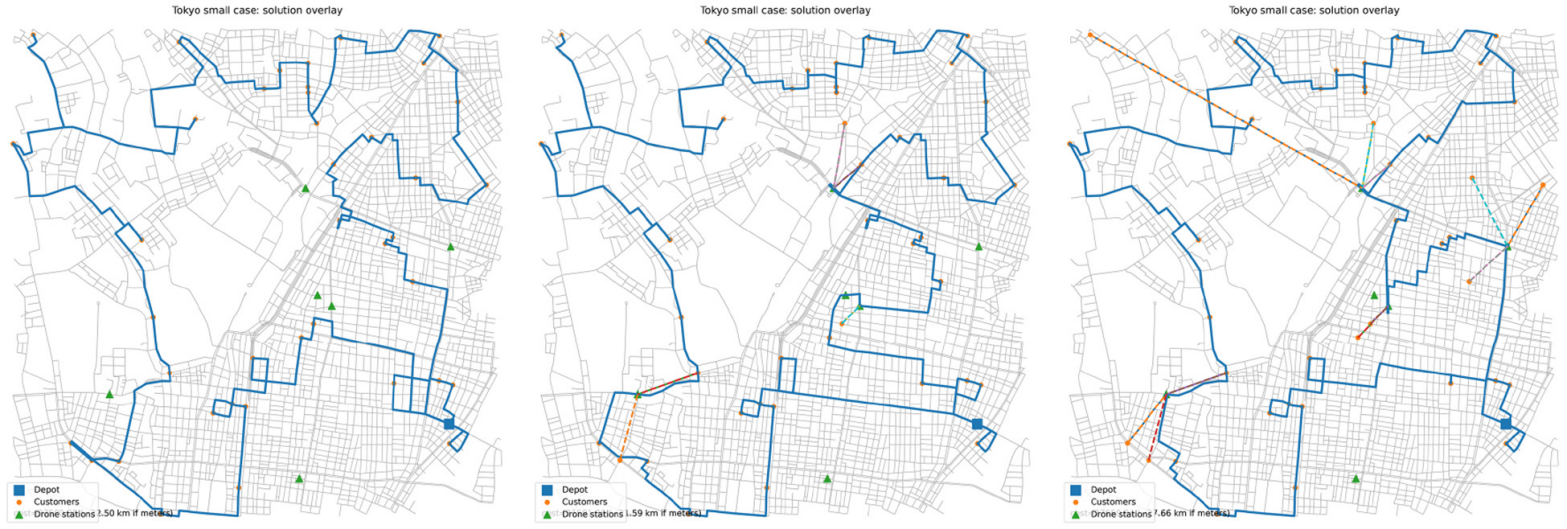
Solution overlay with drone speed 0, 1, and 3/4.

In this section, we download the road network map of the Tokyo metropolitan area and simulate a city-scale last-mile delivery scenario using the proposed F2T-VRP-DS model and the IALNS algorithm. The map considered in this case study covers an area of approximately 5 km × 5 km and includes one depot, 40 customer nodes, and six drone stations. The road network data used in this study are publicly available and are obtained from OpenStreetMap (https://www.openstreetmap.org ). Figure 6 illustrates the resulting delivery routes for the Taito district of Tokyo.

One important difference between real-world maps and Solomon benchmark instances lies in the truck routing structure. In conventional VRP benchmark datasets, the travel distance between customers is approximated by the Euclidean distance between their coordinates, which essentially ignores the constraints imposed by real road networks. However, in real-world urban delivery scenarios, trucks are required to travel along road networks, whereas drones are able to fly directly across the terrain. As a result, the distance calculation logic between nodes differs for these two delivery modes. In this study, we first apply the Dijkstra algorithm to compute the shortest paths between all pairs of nodes, which are then used as the travel distances for trucks. For drones, the distances between nodes are still calculated using the Euclidean distance, implying that drones are assumed to fly in straight lines.

By varying the drone speed, we further investigate the role of drones in the real-world dataset. The results in Table 7 indicate that the use of drones can effectively reduce the total delivery time. However, as the drone speed continues to increase, its marginal contribution to solution quality gradually diminishes. In particular, when the drone speed exceeds 3, no additional customers are assigned to drones, which corroborates the conclusion drawn in the previous section. Moreover, we observe that the proportion of customers served by drones is lower in the real-world dataset. This phenomenon may be attributed to the fact that trucks are required to follow realistic road networks, which increases the travel cost to reach certain drone stations. We believe that the optimal placement of drone launch stations in real urban environments represents a promising direction for future research.

**Table 7:** Solutions of real-world case.

**Drone speed**	**Drone served customers**	***N*_drone_**	**Cost**
0	–	–	32.50
1	20, 23, 28, 30, 39	5	31.59
3	1, 2, 14, 17, 20, 22, 23, 26, 28, 30, 33, 39	11	28.56
4	1, 2, 14, 17, 20, 22, 23, 26, 28, 30, 33, 39	11	27.66

## 6 Conclusion

This study introduces a novel variant of the two-tier vehicle routing problem (2T-VRP), referred to as the Flexible Two-Tier Vehicle Routing Problem with Drone Stations (F2T-VRP-DS). The model integrates two delivery layers—trucks and drones—and differs from conventional 2T-VRP formulations by allowing trucks not only to transport goods to drone stations but also to deliver directly to customers. To address this problem, a MILP formulation was developed, and an enhanced Adaptive Large Neighborhood Search (IALNS) algorithm was proposed to handle large-scale instances efficiently. The proposed IALNS improves upon the classical ALNS framework by incorporating an advanced initial solution based on the Clarke and Wright Savings algorithm and a simulated annealing–based acceptance criterion. Experiments conducted on modified Solomon benchmark instances with added drone stations demonstrate the effectiveness of the proposed approach. For small and medium-sized problems, IALNS achieves an average improvement of 1.34% over MILP and 29.73% over the ACWS initial solution, while for large-scale instances, it surpasses MILP and ACWS by 13.42% and 21.37%, respectively. Particularly, the MILP formulation fails to reach optimality within the time limit for larger cases, whereas IALNS consistently generates high-quality solutions within seconds. Meanwhile, we also compare our approach with the current state-of-the-art GVNS method. The results demonstrate that the proposed algorithm exhibits clear advantages over GVNS in both computational time and solution quality. Furthermore, several sensitivity analyses were performed to investigate the influence of key drone performance parameters on system performance.

Our future work will focus on the following aspects. First, this study does not consider time window constraints. In real urban delivery operations, although self-service pickup is possible, time windows can effectively represent customer availability, which helps improve the efficiency of actual delivery systems. Therefore, we plan to incorporate time window constraints into the model. Second, the multi-depot (MD) problem is also a classical variant of the VRP, and we will consider its application to the F2T-VRP. Finally, we observed that although the IALNS algorithm performs much faster than the MILP model, its performance on small instances is slightly inferior, possibly due to getting trapped in local optima. Hence, we plan to further improve the algorithm to enhance its performance and stability.

## Data Availability

The raw data supporting the conclusions of this article will be made available by the authors, without undue reservation.

## References

[B1] Alamouri A. Lampert A. Gerke M. (2023). Impact of drone regulations on drone use in geospatial applications and research: focus on visual range conditions, geofencing and privacy considerations. PFG-J. Photogram. Remote Sens. Geoinform. Sci. 91, 381–389. doi: 10.1007/s41064-023-00246-y

[B2] Alfandari L. Ljubić I. Da Silva M. D. M. (2022). A tailored benders decomposition approach for last-mile delivery with autonomous robots. Eur. J. Operat. Res. 299, 510–525. doi: 10.1016/j.ejor.2021.06.048

[B3] Altınel İ. K. Öncan T. (2005). A new enhancement of the clarke and wright savings heuristic for the capacitated vehicle routing problem. J. Operat. Res. Soc. 56, 954–961. doi: 10.1057/palgrave.jors.2601916

[B4] Bogyrbayeva A. Meraliyev M. Mustakhov T. Dauletbayev B. (2024). Machine learning to solve vehicle routing problems: a survey. IEEE Trans. Intell. Transport. Syst. 25, 4754–4772. doi: 10.1109/TITS.2023.3334976

[B5] Campuzano G. Lalla-Ruiz E. Mes M. (2025). The two-tier multi-depot vehicle routing problem with robot stations and time windows. Eng. Applic. Artif. Intell. 147:110258. doi: 10.1016/j.engappai.2025.110258

[B6] Che A. Wang W. Mu X. Zhang Y. Feng J. (2022). Tabu-based adaptive large neighborhood search for multi-depot petrol station replenishment with open inter-depot routes. IEEE Trans. Intell. Transp. Syst. 24, 316–330. doi: 10.1109/TITS.2022.3215084

[B7] Chen C. Demir E. Huang Y. (2021). An adaptive large neighborhood search heuristic for the vehicle routing problem with time windows and delivery robots. Eur. J. Operat. Res. 294, 1164–1180. doi: 10.1016/j.ejor.2021.02.027

[B8] Cho Y. H. Baek D. Chen Y. Jung M. J. Vinco S. Macii E. . (2022). Multi-criteria coordinated electric vehicle-drone hybrid delivery service planning. IEEE Trans. Vehic. Technol. 72, 5892–5905. doi: 10.1109/TVT.2022.3232799

[B9] Crainic T. G. Ricciardi N. Storchi G. (2009). Models for evaluating and planning city logistics systems. Transp. Sci. 43, 432–454. doi: 10.1287/trsc.1090.0279

[B10] De Freitas J. C. Penna P. H. V. (2020). A variable neighborhood search for flying sidekick traveling salesman problem. Int. Trans. Operat. Res. 27, 267–290. doi: 10.1111/itor.12671

[B11] El-Adle A. M. Ghoniem A. Haouari M. (2023). A variable neighborhood search for parcel delivery by vehicle with drone cycles. Comput. Operat. Res. 159:106319. doi: 10.1016/j.cor.2023.106319

[B12] Franceschetti A. Demir E. Honhon D. Van Woensel T. Laporte G. Stobbe M. (2017). A metaheuristic for the time-dependent pollution-routing problem. Eur. J. Operat. Res. 259, 972–991. doi: 10.1016/j.ejor.2016.11.026

[B13] Gao J. Zhen L. Laporte G. He X. (2023). Scheduling trucks and drones for cooperative deliveries. Transp. Res. Part E: Logist. Transp. Rev. 178:103267. doi: 10.1016/j.tre.2023.103267

[B14] Heimfarth A. Ostermeier M. Hübner A. (2022). A mixed truck and robot delivery approach for the daily supply of customers. Eur. J. Operat. Res. 303, 401–421. doi: 10.1016/j.ejor.2022.02.028

[B15] Huang S.-H. Huang Y.-H. Blazquez C. A. Chen C.-Y. (2022). Solving the vehicle routing problem with drone for delivery services using an ant colony optimization algorithm. Adv. Eng. Inform. 51:101536. doi: 10.1016/j.aei.2022.101536

[B16] Jeong H. Y. Song B. D. Lee S. (2019). Truck-drone hybrid delivery routing: payload-energy dependency and no-fly zones. Int. J. Prod. Econ. 214, 220–233. doi: 10.1016/j.ijpe.2019.01.010

[B17] Kong J. Xie M. Wang H. (2025). Integrating autonomous vehicles and drones for last-mile delivery: a routing problem with two types of drones and multiple visits. Drones 9:280. doi: 10.3390/drones9040280

[B18] Kuo R. Lu S.-H. Lai P.-Y. Mara S. T. W. (2022). Vehicle routing problem with drones considering time windows. Expert Syst. Applic. 191:116264. doi: 10.1016/j.eswa.2021.116264

[B19] Li Y. Zhang Y. Li Y. Zhu X. (2025). Vehicle routing problem with drones and variable service times for agricultural virus monitoring. Eur. J. Oper. Res. 331, 520–533. doi: 10.1016/j.ejor.2025.09.021

[B20] Liang Y. Luo H. Duan H. Li D. Liao H. Feng J. . (2024). Meituan's real-time intelligent dispatching algorithms build the world's largest minute-level delivery network. INFORMS J. Appl. Anal. 54, 84–101. doi: 10.1287/inte.2023.0084

[B21] Lyu M. Liu W. He Q. Wu L. (2025). Urban food delivery service optimisation with coordinated delivery riders and drones. Transp. Res. Part E: Logist. Transp. Rev. 204:104412. doi: 10.1016/j.tre.2025.104412

[B22] Mahmoudi B. Eshghi K. (2025). The multi-visit split delivery vrp with drones considering en-route launches and rendezvouses: application to post-disaster relief operations. Comput. Indus. Eng. 206:111232. doi: 10.1016/j.cie.2025.111232

[B23] Morim A. Campuzano G. Amorim P. Mes M. Lalla-Ruiz E. (2024). The drone-assisted vehicle routing problem with robot stations. Expert Syst. Applic. 238:121741. doi: 10.1016/j.eswa.2023.121741

[B24] Murray C. C. Chu A. G. (2015). The flying sidekick traveling salesman problem: optimization of drone-assisted parcel delivery. Transp. Res. Part C: Emerg. Technol. 54, 86–109. doi: 10.1016/j.trc.2015.03.005

[B25] Murray C. C. Raj R. (2020). The multiple flying sidekicks traveling salesman problem: parcel delivery with multiple drones. Transp. Res. Part C: Emerg. Technol. 110:368–398. doi: 10.1016/j.trc.2019.11.003

[B26] Perboli G. Tadei R. Vigo D. (2011). The two-echelon capacitated vehicle routing problem: models and math-based heuristics. Transp. Sci. 45, 364–380. doi: 10.1287/trsc.1110.0368

[B27] Poeting M. Schaudt S. Clausen U. (2019). “A comprehensive case study in last-mile delivery concepts for parcel robots,” in 2019 Winter Simulation Conference (WSC) (National Harbor, MD: IEEE), 1779–1788. doi: 10.1109/WSC40007.2019.9004811

[B28] Rave A. Fontaine P. Kuhn H. (2023). Drone location and vehicle fleet planning with trucks and aerial drones. Eur. J. Oper. Res. 308, 113–130. doi: 10.1016/j.ejor.2022.10.015

[B29] Rojas Viloria D. Solano-Charris E. L. Muñoz-Villamizar A. Montoya-Torres J. R. (2021). Unmanned aerial vehicles/drones in vehicle routing problems: a literature review. Int. Trans. Oper. Res. 28, 1626–1657. doi: 10.1111/itor.12783

[B30] Ropke S. Pisinger D. (2006). An adaptive large neighborhood search heuristic for the pickup and delivery problem with time windows. Transp. Sci. 40, 455–472. doi: 10.1287/trsc.1050.0135

[B31] Sacramento D. Pisinger D. Ropke S. (2019). An adaptive large neighborhood search metaheuristic for the vehicle routing problem with drones. Transp. Res. Part C: Emerg. Technol. 102, 289–315. doi: 10.1016/j.trc.2019.02.018

[B32] Shaw P. (1998). “Using constraint programming and local search methods to solve vehicle routing problems,” in International Conference on Principles and Practice of Constraint Programming (Berlin: Springer), 417–431. doi: 10.1007/3-540-49481-2_30

[B33] Sluijk N. Florio A. M. Kinable J. Dellaert N. Van Woensel T. (2023). Two-echelon vehicle routing problems: a literature review. Eur. J. Operat. Res. 304, 865–886. doi: 10.1016/j.ejor.2022.02.022

[B34] Tamke F. Buscher U. (2023). The vehicle routing problem with drones and drone speed selection. Comput. Operat. Res. 152:106112. doi: 10.1016/j.cor.2022.106112

[B35] Thibbotuwawa A. Bocewicz G. Nielsen P. Banaszak Z. (2020). Unmanned aerial vehicle routing problems: a literature review. Appl. Sci. 10:4504. doi: 10.3390/app10134504

[B36] Toaza B. Esztergár-Kiss D. (2023). A review of metaheuristic algorithms for solving tsp-based scheduling optimization problems. Appl. Soft Comput. 148:110908. doi: 10.1016/j.asoc.2023.110908

[B37] Tu P. A. Dat N. T. Dung P. Q. (2018). “Traveling salesman problem with multiple drones,” in Proceedings of the 9th International Symposium on Information and Communication Technology (New York, NY: Association for Computing Machinery), 46–53.

[B38] Wang D. Hu P. Du J. Zhou P. Deng T. Hu M. (2019). Routing and scheduling for hybrid truck-drone collaborative parcel delivery with independent and truck-carried drones. IEEE Internet Things J. 6, 10483–10495. doi: 10.1109/JIOT.2019.2939397

[B39] Wang Z. Sheu J.-B. (2019). Vehicle routing problem with drones. Transp. Res. Part B: Methodol. 122, 350–364. doi: 10.1016/j.trb.2019.03.005

[B40] Yu S. Puchinger J. Sun S. (2020). Two-echelon urban deliveries using autonomous vehicles. Transp. Res. Part E: Logist. Transp. Rev. 141:102018. doi: 10.1016/j.tre.2020.102018

[B41] Zhou J. Yi J. Yang Z. Pu H. Li X. Luo J. (2025). A survey on vehicle-drone cooperative delivery operations optimization: models, methods, and future research directions. Swarm Evol. Comput. 92:101780. doi: 10.1016/j.swevo.2024.101780

